# Draft Genome Sequences of the Aerobic Strains* Lactobacillus gasseri* AL3 and AL5

**DOI:** 10.1128/genomeA.00213-17

**Published:** 2017-05-04

**Authors:** Diamante Maresca, Francesca De Filippis, Hanne L. P. Tytgat, Willem M. de Vos, Gianluigi Mauriello

**Affiliations:** a Department of Agricultural Sciences, University of Naples Federico II, Portici, Italy; b Laboratory of Microbiology, Wageningen University, Wageningen, The Netherlands; c Department of Bacteriology and Immunology, University of Helsinki, RPU Immunobiology, Haartman Institute, Helsinki, Finland

## Abstract

Adaptation to the aerobic environment has been investigated in heterofermentative lactobacilli, while data on how homofermentative lactobacilli adapt to oxygen are limited. We report here the draft genome sequences of the aerobic strains *Lactobacillus gasseri* AL3 and AL5 that allow an in-depth investigation of the genes involved in oxidative metabolism and the stress response.

## GENOME ANNOUNCEMENT

*Lactobacillus gasseri* is abundantly present in the vaginal microbiota, is found in the human intestine, and is receiving particular attention due to its industrial applications (1–5). *Lactobacillus gasseri* strains AL3 and AL5, previously isolated from infant feces at the University of Naples, were selected for their ability to grow under respiratory-promoting conditions and to scavenge hydrogen peroxide and reactive oxygen species. To gain a better understanding of the genetic basis of the observed phenotypic features, whole-genome sequencing was carried out. Strains were grown on de Man-Rogosa-Sharpe (MRS) broth, and pellets were sent for genomic DNA extraction and sequencing on an Illumina HiSeq platform at the GATC Biotech AG Company (Germany). Sequencing led to 11,092,500 (AL3) and 12,136,300 (AL5) paired-end reads. Low-quality bases (Phred score <20) were trimmed, and reads shorter than 60 bp were discarded using the SolexaQA++ software (6). Reads were assembled using IDBA version 1.1.1 (7), and assembly was improved by using SSPACE (8). Genes were predicted with PROKKA (9). Predicted genes were queried in the NCBI database against proteins involved in aerobic (pyruvate oxidase [POX], lactate oxidase [LOX], NADH oxidase [NOX], and acetate kinase [ACK]), respiratory metabolism (NADH dehydrogenase [NDH], ubiquinone/menaquinone biosynthesis C-methylase [UbiE], cytochrome *bd*-I oxidase [CydABCD]), and oxidative stress response (NADH-peroxidase [NPR], glutathione reductase [GR], glutathione peroxidase [GOP], γ-glutamylcystiene synthetase [GshA], glutathione synthetase [GshB], bifunctional glutamate-cysteine ligase/glutathione synthetase [GshF], thioredoxin reductase [TrxR], thioredoxin peroxidase [TrxP], superoxide dismutase [SOD], catalase [CAT], pseudocatalase [Man-CAT], and DNA-binding protein from starved cells [Dps]). A match was considered valid when showing more than 40% of identity over at least 90% of the query length. Functional annotation of all genes was carried out using the NCBI Prokaryotic Genome Annotation Pipeline (PGAP). The presence of *ribA* and *ribH* genes was used as a *Lactobacillus gasseri* species-specific marker (10). The AL3 and AL5 draft genomes have total lengths of 1,994,887 and 1,999,212 bp, respectively. The AL3 draft genome contains 16 scaffolds (average coverage, 1,329×), with an overall G+C content of 34.81%. The AL5 draft genome contains 35 scaffolds (average coverage, 583×), with an overall G+C content of 34.87% (*N*_50_, 1,125,240 bp; *N*_75_, 571,948 bp). A total of 1,945 and 1,998 genes were found in the AL3 and AL5 genomes, respectively. The AL3 draft genome contains 1,874 predicted coding sequences (CDSs), 77 pseudogenes, 61 tRNAs, and 71 rRNAs. The AL5 draft genome contains 1,932 CDSs, 100 pseudogenes, 56 tRNAs, and 66 rRNAs. Plasmids were not found by using PlasmidFinder 1.3 (11). We found genes with 98 to 100% identity with genes encoding POX, LOX, ACK, NDH, UbiE, CydABCD, NPR, GR, TrxA, and TrxB proteins previously identified in other *Lactobacillus* genomes. Moreover, both strains show genes with high homology (ranging from 40 to 73%) with genes encoding NOX of *Lactobacillus brevis*, GshA of *Micrococcus luteus*, SOD of *Atopostipes suicloacalis*, and Dps of *Lactobacillus reuteri*. Genes encoding GshB, GshF, CAT, and Man-CAT were not found.

### Accession number(s).

This whole-genome shotgun project has been deposited at DDBJ/ENA/GenBank under the accession numbers MTZT00000000 and MUJA00000000 for strains AL3 and AL5, respectively. The versions described in this paper are versions MTZT00000000 and MUJA01000000.
